# Microbial Ecology on Solar Panels in Berkeley, CA, United States

**DOI:** 10.3389/fmicb.2018.03043

**Published:** 2018-12-11

**Authors:** Manuel Porcar, Katherine B. Louie, Suzanne M. Kosina, Marc W. Van Goethem, Benjamin P. Bowen, Kristie Tanner, Trent R. Northen

**Affiliations:** ^1^Institute for Integrative Systems Biology (I2SysBio), University of Valencia-CSIC, Paterna, Spain; ^2^Darwin Bioprospecting Excellence S.L., Parc Científic de la Universitat de València, Paterna, Spain; ^3^Lawrence Berkeley National Laboratory, Joint Genome Institute, Walnut Creek, CA, United States; ^4^Environmental Genomics and Systems Biology Division, Lawrence Berkeley National Laboratory, Berkeley, CA, United States

**Keywords:** solar panels, microbiome, metabolomics, metagenomics, stress-resistant bacteria

## Abstract

Solar panels can be found practically all over the world and represent a standard surface that can be colonized by microbial communities that are resistant to harsh environmental conditions, including high irradiation, temperature fluctuations and desiccation. These properties make them not only ideal sources of stress-resistant bacteria, but also standard devices to study the microbial communities and their colonization process from different areas of Earth. We report here a comprehensive description of the microbial communities associated with solar panels in Berkeley, CA, United States. Cultivable bacteria were isolated to characterize their adhesive capabilities, and UV- and desiccation-resistance properties. Furthermore, a parallel culture-independent metagenomic and metabolomic approach has allowed us to gain insight on the taxonomic and functional nature of these communities. Metagenomic analysis was performed using the Illumina HiSeq2500 sequencing platform, revealing that the bacterial population of the Berkeley solar panels is composed mainly of Actinobacteria, Bacteroidetes and Proteobacteria, as well as lower amounts of Deinococcus-Thermus and Firmicutes. Furthermore, a clear predominance of *Hymenobacter* sp. was also observed. A functional analysis revealed that pathways involved in the persistence of microbes on solar panels (i.e., stress response, capsule development, and metabolite repair) and genes assigned to carotenoid biosynthesis were common to all metagenomes. On the other hand, genes involved in photosynthetic pathways and general autotrophic subsystems were rare, suggesting that these pathways are not critical for persistence on solar panels. Metabolomics was performed using a liquid chromatography tandem mass spectrometry (LC-MS/MS) approach. When comparing the metabolome of the solar panels from Berkeley and from Valencia (Spain), a very similar composition in polar metabolites could be observed, although some metabolites appeared to be differentially represented (for example, trigonelline, pantolactone and 5-valerolactone were more abundant in the samples from Valencia than in the ones from Berkeley). Furthermore, triglyceride metabolites were highly abundant in all the solar panel samples, and both locations displayed similar profiles. The comparison of the taxonomic profile of the Californian solar panels with those previously described in Spain revealed striking similarities, highlighting the central role of both selective pressures and the ubiquity of microbial populations in the colonization and establishment of microbial communities.

## Introduction

It has recently been calculated that there might be as many as one trillion different species on Earth, the vast majority of which are microorganisms (Locey and Lennon, 2016). Microorganisms are ubiquitous, and can even be found in extreme environments such as thermal springs (Kizilova et al., 2014), marine trenches (Felden et al., 2014) and man-made structures (Vilanova et al., 2015). Interestingly, solar panels have been reported to harbor a diverse microbial community, mainly composed of desiccation/irradiation-adapted microorganisms, similar to those found in other highly irradiated environments, such as deserts, plant surfaces and polar microbial mats (Dorado-Morales et al., 2016; Tanner et al., 2018). The presence of biofilms on the surface of photovoltaic panels from Brazil has been shown to decrease the efficiency by 11% after 18 months (Shirakawa et al., 2015). Moreover, dust particle accumulation during drought seasons (a process known as “soiling”) has been associated with a decrease in the yield of California photovoltaic panels, accounting for a loss of up to 0.1% of the power production per day (Mejia and Kleissl, 2013). Biofilm and dust accumulation on outdoor glass surfaces such as photovoltaic panels depend, among other factors, on the coating and angle (Mejia and Kleissl, 2013; Banerjee et al., 2015). Although the effect of biofilms on soiling in solar panels has not been quantified, it seems reasonable to hypothesize that biofilm growth might increase dust adhesion. Despite the economic benefits of understanding the association between the decreased yield of solar panels and the biofilms formed on them, little is known about how the latitude, climate, the physical characteristics of the panels affect the microbial communities in this still poorly characterized ecological niche.

Solar panels represent a particularly interesting environment due to their simple, yet standard structure and orientation (an equator-facing glass surface); their abundance worldwide; and the fact that these inert, non-porous bidimensional artificial surfaces are a proxy of sun-exposed natural environments such as rocks, the phyllosphere or the top layer of biological soil crusts. A previous study assessing the microbiome of solar panels from the North and South Poles revealed that despite the geographical distance between both environments, the composition of the solar panel microbiome is very similar (Tanner et al., 2018). Furthermore, solar panel surfaces can be used as sources for the isolation of interesting radiation- and desiccation-resistant bacteria. A study by Ragon et al. (2011) revealed that biofilms growing on sunlight-exposed surfaces are naturally resistant to Chernobyl ionizing-radiation levels which is due to their natural adaptation to periodical desiccation and UV-irradiation. Survival of ionizing radiation- and desiccation-resistant bacteria has been previously attributed to the ability of these microorganisms to protect their proteins from the oxidative damage generated during irradiation, leading to functioning repair systems that work more efficiently during recovery than those in bacteria that are sensitive to radiation (Fredrickson et al., 2008).

A previous description of the microbial community on solar panels from the Mediterranean city of Valencia, Spain revealed the presence of black fungi, some phototrophs and a surprising diversity of sun-adapted bacterial taxa, dominated by *Hymenobacter* spp., *Sphingomonas* spp., and *Deinococcus* spp. (Dorado-Morales et al., 2016). In order to shed light on the ecology of the solar panel microbiome and to further compare the microbial profiles on panels from distant geographical locations, we present here a comprehensive characterization of the microbial communities of solar panels in another coastal city distant from Valencia: Berkeley, CA, United States. Both cities share a Mediterranean climate, a relatively high humidity and a protracted dry summer season. They are also at similar altitudes and latitudes (Berkeley is less than two degrees south from Valencia: 37° 52′ and 39° 28′, respectively) and thus receive similar annual UV irradiation doses. In the present work, we have analyzed the functional and taxonomic diversity of the solar panels of the University of California in Berkeley through metagenomics; compared the microbial communities with those described on solar panels from Valencia (Dorado-Morales et al., 2016); identified several key compounds of its metabolome through mass spectrometry; and studied the adhesion, irradiation and desiccation resistance abilities of selected cultivable isolates in the laboratory.

## Materials and Methods

### Sampling

Sampling was carried out in August 2016 on the Lawrence Berkeley National Laboratory main campus (Berkeley, CA, United States). Three independent, adjacent photovoltaic solar panels of building 30 (installed and uncleaned for at least 18 months) were sampled by pouring sterile PBS on the surface and by strongly scraping the surface with autoclave-sterilized T-shaped rubber and steel window cleaners (squeegees). Approximately 40 mL of soil panel dust slurry was collected from each solar panel using sterile pipettes, transferred into sterile polypropylene conical tubes and immediately transported to the laboratory for further processing. There, aliquots were taken for culturing and colonization experiments, and the remaining volume was split in two, centrifuged and the pellets stored at -80°C until required for metagenomic and metabolic analysis. The solar panels from Valencia (Spain) were sampled using the same procedure, obtaining a final volume of 5 mL that was sent on dry ice to the laboratory in Berkeley, CA, United States, for metabolomics analysis. The metagenomic sequences obtained in the previous report by Dorado-Morales et al. (2016) were used for the taxonomic comparison between the Spanish and Californian solar panels.

### Culture Media and Conditions

A total of 300 μL aliquots of each sample were transferred into sterile 1.5 mL microcentrifuge tubes and let stand for 5 min at room temperature prior to spreading 50 μL of the supernatant on freshly prepared LB and R2A agar plates. A dual approach with nutrient-rich (LB) and nutrient-poor (R2A) media was used in order to allow microorganisms with different nutrient requirements to grow. All cultures were performed in duplicate and incubated at 4°C, room temperature (RT) (∼22°C), 27 and 50°C for 22, 9, 5, and 3 days, respectively. Selected colonies corresponding to the most frequent phenotypes (i.e., light pink) on R2A were re-streaked on fresh R2A plates and pure cultures grown on solid medium were cryopreserved in 25% glycerol.

Pooled aliquots (10 μL) of the three samples were placed on microscope slides (VWR CAT No. 48393048, 22X40 mm) and dried at room temperature (RT) under sterile conditions. The slides were then washed with sterile water, dried again and subjected to 2 min of UV irradiation in the hood and at a distance of 46 cm from the UV light (Air Clean 600 PCR workstation equipped with a 254 nm short-wave UV light). The dried and irradiated microscope slides were kept in the hood at RT for 30 min and then transferred sample side down onto the surface of R2A agar plates, where they settled for 30 min before being removed. Plates were incubated at RT for 4 days. Surviving colonies, as well as the ten non-irradiated isolates selected among those growing in R2A plates were selected for further studies. Colonies were identified through amplification and sequencing of almost the full-length 16S rRNA gene (in exception of a small fragment of ∼200 base pairs at the beginning of the V1 regions) through Sanger sequencing, followed by a taxonomic assignment using the NCBI Blast Tool. All but one of the sequences displayed 98–99% similarity with the closest match. The exception was an isolate belonging to the *Deinococcus* genus, which displayed 96% similarity with the closest match.

### Colonization Experiments

A loopful of each selected isolate, grown for 1 week on R2A agar at room temperature, was suspended in liquid R2A and optical densities (600 nm) were adjusted to 0.1 absorbance units. A 10 μL droplet of each suspension was placed on a sterile glass slide and kept at RT for 1 h. Then, 10 μL of R2A were added to each droplet to prevent desiccation and the assay was continued for one more hour, after which droplets were removed by washing the slides three times with 1 mL of sterile water. The slides were allowed to completely dry in the hood for 1 h and were then either placed sample side down on the surface of R2A plates (glass colonization assay); subjected to UV irradiation (UV-resistance assay); or subjected to 72 h of further desiccation at RT (desiccation-resistance assay).

For the glass colonization assay, the slides were placed on solid R2A medium and incubated for 30 min at RT to allow for transfer of the bacteria to the solid medium. Then, the glass slides were removed and the plates were incubated at RT for 4 days. For the UV resistance assays, after washing and drying the slides (as described above), the 14 selected isolates were subjected to 2 min of irradiation with the UV lamp in the Air Clean 600 PCR workstation and at 15 cm distance from the lamp. UV-treated glass slides were placed on R2A agar plates and incubated as described above (30 min at room temperature) to allow the transfer of the bacteria. Finally, the desiccation-resistance assays were carried out with the 14 selected strains as described above (without UV irradiation) by air-drying washed droplets for 72 h inside the hood prior to transferring them to R2A plates, where they were incubated for 30 min at room temperature to allow transfer of the bacteria.

### DNA Isolation and Metagenomic Analysis

Metagenomic DNA was isolated from solar panels samples as previously described (Dorado-Morales et al., 2016). Briefly, pellets were thawed on ice, incubated with lysozyme in the PowerBead tubes solution without the beads (PowerSoil, MoBio) at 37°C for 10 min, and then transferred back to the PowerBead tubes containing the beads. The extraction was continued following the instructions of the manufacturer.

Metagenomic analysis and annotations were performed as follows. For the library construction, 10 ng of DNA was sheared to 300 bp using the Covaris LE220 (Covaris) and size selected using SPRI beads (Beckman Coulter). The fragments were treated with end-repair, A-tailing, and ligation of Illumina compatible adapters (IDT, Inc), and 5 cycles of PCR was used to enrich for the final library. The libraries were quantified and run on a Roche LightCycler 480 real-time PCR instrument, followed by preparation for sequencing on the Illumina HiSeq2500 sequencing platform using a TruSeq Rapid paired-end cluster kit, v.4. After sequencing, known Illumina adapters were removed and the reads were then processed using BBDuk filtering and trimming (where quality values were less than 12). Remaining reads were mapped to a masked version of human HG19 with BBMap, discarding all hits over 93% identity. Trimmed, screened, paired-end Illumina reads were assembled using megahit assembler using a range of Kmers (Li et al., 2015). The entire read set output from the previously described read pre-processing step were mapped to the final assembly and coverage information generated using BBMap. Annotation was performed using the DOE-JGI Metagenome Annotation Pipeline (MAP v.4) (Huntemann et al., 2016). Open reading frames (ORFs) were identified from each of the three assemblies using Prodigal v.2.6.3 software (Hyatt et al., 2010). Genes were subsequently annotated against the entire NCBI nr-database using DIAMOND (Buchfink et al., 2014).

Taxonomic information was obtained from the metagenomic data using the microbial classification engine “Centrifuge” (Kim et al., 2016), as well as the aforementioned NCBI non-redundant database. Taxonomic and functional affiliations were visualized in the MEGAN6 software environment (Huson et al., 2007). For comparison of solar panels from different locations, a radial tree representing phylogenetic distances between solar panels from Berkeley, CA, United States and Valencia, Spain was constructed using the JGI IMG/MER database tools, with a percent identity above 90%. Statistical analyses were performed both using STAMP (Parks et al., 2014) and in the R statistical environment.

### Metabolite Extractions

Solar panel slurry pellets were collected by centrifugation of 5 mL (Valencia, Spain) or 10 mL (Berkeley, CA, United States) of solar panel dust slurry (2655 RCF for 5 min). Empty tubes were included as extraction controls to account for ions resulting from procedural methods.

For extraction of hydrophilic metabolites, the slurry pellets were extracted in methanol. Briefly, the pellets were resuspended in 2 mL of 100% methanol, vortexed for 10 s, sonicated for 20 min. in an ice bath, and then incubated at 4°C overnight. The following day, the methanol solutions were vortexed again and centrifuged at 6000 RCF for 3 min to pellet insoluble material. The supernatants were then dried under vacuum at room temperature for 6 h (Thermo SpeedVac Concentration and Trap) which each yielded ∼10 μL of viscous yellow fluid. These were then resuspended in 150 μL of methanol with internal standards. The resuspensions were vortexed 10 s, sonicated 20 min in an ice bath and centrifuged at 6000 RCF for 3 min to pellet insoluble material; supernatants were filtered through a 0.22 μm microcentrifuge filtration devices (Pall, ODM02C34) and filtrates were transferred to glass vials for analysis. The internal standard mix used for the Valencia, Spain sample was a 2000-fold dilution of universally labeled 15N, 13C amino acid mix (Sigma, 767964). The internal standards used for the Berkeley, CA, United States samples included 1 μg/mL 2-amino-3-bromo-5-methylbenzoic acid (Sigma R435902), 5 μg/mL 3,6-dihydroxy-4-methylpyridazine (Sigma 668141), 5 μg/mL 13C-15N-L-phenylalanine (Sigma 608017), 10 μg/mL d4-lysine (Sigma 616192), 10 μg/mL d5-benzoic acid (Sigma 217158), and 2 μg/mL 9-anthracene carboxylic acid (Sigma A89405).

For triglycerides, chloroform extractions were performed on slurry pellets (collected as described above) using a modified Bligh-Dyer approach (Bligh and Dyer, 1959). Briefly, 120 μL of water was added to each pellet, vortexed, then 450 μL of 2:1 MeOH:CH_3_Cl was added for a final ratio of 2:1:0.8 MeOH:CH_3_Cl:H_2_O followed by a brief vortex and incubation for 15 min in a sonicating water bath. An additional 150 μL CH_3_Cl and 150 μL H_2_O was added to create a final ratio of 1:1:0.9 MeOH:CH_3_Cl:H_2_O, then briefly vortexed and incubated for 10 min in a sonicating water bath. After centrifuging samples for 2 min at 2655 RCF, the lower lipid-enriched chloroform phase was transferred to a new tube. 300 μL of chloroform was then added to the remaining pellet (methanol-water layer), followed by repeat sonication and centrifugation, and the bottom chloroform phase was combined with the previously collected extract. Chloroform extracts of lipid were then dried in a SpeedVac (SPD111V, Thermo Scientific) and stored at -20°C. Prior to analysis, dried extracts were resuspended in 3:3:4 isopropanol:acetonitrile:methanol (IPA:ACN:MeOH), centrifuge-filtered through a 0.22 μm PVDF membrane (Millipore Ultrafree-MC) containing an internal standard mixture of 1 μg/mL 2-Amino-3-bromo-5-methylbenzoic acid (ABMBA) and 4 μM each of deuterated lipids including: 17:0-17:1-17:0 D5 triglyceride (Avanti 110544), 18:0-18:1 D5 phosphoglyceride (Avanti 110899), D9 oleic acid (Avanti 850809O), 1,3-16:1 D5 diglyceride (Avanti 110579), and dipalmitoyl glycerol trimethyl homoserine D9 (Avanti 857463). Filtrates were transferred to glass vials for analysis.

### Liquid Chromatography Tandem Mass Spectrometry (LC-MS/MS) Based Metabolomics

Chromatographic separations were performed using an Agilent 1290 LC stack, with MS and MS/MS data collected using a Q Exactive hybrid Quadrupole-Orbitrap Mass Spectrometer equipped with a heated electrospray ionization (HESI-II) source probe (Thermo Scientific, San Jose, CA, United States). All chemicals and solvents were of LCMS or HPLC grade.

Polar metabolites were chromatographically separated using a 5 μm, 150 × 2.1 mm, 200Å ZIC-HILIC column containing sulfobetaine (zwitterionic) silica based stationary phase (Merck Millipore) under the following conditions: 0.45 mL/min. flow rate, 40°C column temperature, and a 2 μL injection volume. Mobile phases (A: 5 mM ammonium acetate in water, and B: 5 mM ammonium acetate, 95% v/v acetonitrile in water) were varied as follows: 1.5 min hold at 100% B, 13.5 min linear gradient to 65% B, 3 min linear gradient to 0% B, 5 min hold at 0% B, 2 min gradient to 100% B, and a 5 min reequilibration at 100% B.

Triglycerides were chromatographically separated using a 1.8 μm, 50 × 2.1 mm C18 column (Agilent ZORBAX Eclipse Plus C18, Rapid Resolution HD) under the following conditions: 0.4 mL/min flow rate, 55°C column temperature, and a 2 μL injection volume. Mobile phases (A: 40:60 water:acetonitrile with 5 mM ammonium acetate and 0.1% v/v formic acid, and B: 90:10 isopropanol:acetonitrile with 5 mM ammonium acetate and 0.1% v/v formic acid) were varied as follows: 1.5 min hold at 20% B, 2.5 min linear gradient to 55% B, 6 min linear gradient to 80% B, 2 min hold at 80% B, 1.5 min linear gradient to 100% B, 3.5 min hold at 100% B, 1.5 min linear gradient to 20% B and 1.5 min re-equilibration at 20% B.

For all chromatographies, eluted compounds were detected via ESI-MS/MS using the Q Exactive’s data dependent MS2 Top2 function, where the two highest abundance precursor ions reaching at least 1e3 ions within the max ion transfer time (excluding ions with assigned charge ≥4) and not already fragmented in the previous 10 s are selected from a full MS pre-scan from m/z 70–1050 (HILIC) or 80–1200 (C18) at 70,000 resolution with an automatic gain control (AGC) target at 3e6 and 100 millisecond maximum ion transmission, followed by sequential MS/MS fragmentation of each of the two precursors with stepped normalized collision energies (stepped NCE) of 10, 20, and 30 (HILIC) or 10, 20, 40 (C18) at 17,000 resolution with an isolation window of 2 m/z and AGC target at 1e5 and 50 milliseconds; all spectra were stored in centroid data format. The source was set with the sheath gas flow at 55 (arbitrary units), aux gas flow at 20 (arbitrary units), sweep gas flow at 2 (arbitrary units), spray voltage at 3 |kV|, and capillary temperature at 400°C. Internal standards were used for quality control purposes.

### Metabolomics Data Analysis

For HILIC data analysis, retention and fragmentation data were compared to a library of pure reference standards analyzed under the same conditions. MS/MS fragmentation spectra, if collected for the compound of interest, were compared to internal and online spectral databases to confirm identification. A subset of the library was analyzed (as external standards) at the same time as the samples and used for generation of the theoretical retention times using linear regression (to account for retention shifts due to changes in tubing length, mobile phase batches and different lots of column from the manufacturer). Exact mass (+/- 25 ppm at peak apex) and retention time (+/- 0.5 min from theoretical) coupled with MS/MS fragmentation spectra were used to identify compounds with a python-based metabolite atlas analysis (Bowen and Northen, 2010; Yao et al., 2015). Python code is available at https://github.com/biorack/metatlas.

Exact mass and retention time coupled with MS/MS fragmentation spectra were used to identify lipids. Lipid class was determined based on characteristic fragment ions or neutral loss, and coupled with exact mass to determine specific lipid identity (number of carbons in fatty acid tails and degree of unsaturation). In positive ion mode, triglycerides ionized as a singly charged ammonium adduct with fatty acid tails detected in the MS/MS fragmentation spectra (McAnoy et al., 2005). Deuterated TG internal standard was used to verify fragmentation pattern and retention time range for the TG lipid class.

### Availability of Data

Raw and processed data are available on the JGI Genome Portal: https://genome.jgi.doe.gov/portal/solcelcoanalysisunder proposal 503162 “solar cell community analysis.” Metabolomic results from solar panels in Berkeley and Valencia have been deposited under project ID 1196772. The metagenomics from the three Berkeley, CA, United States solar panel communities are available under project IDs: 1123560, 1123562, and 1123564.

## Results

### Cultivable Isolates and Colonization Experiments

**FIGURE 1:**
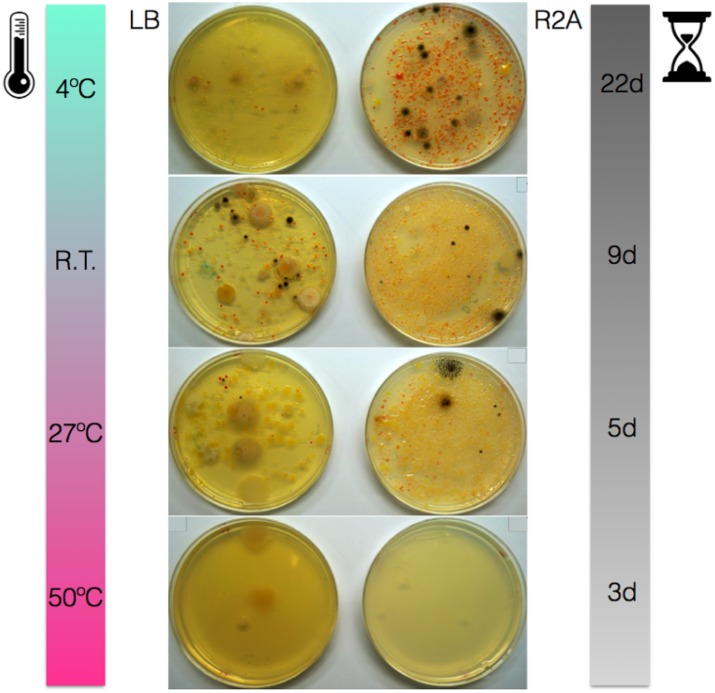
Solar panel samples grown on LB and R2A media and incubated at 4°C, room temperature (22°C), 27 and 50°C for 22, 9, 5, and 3 days, respectively.

Solar panels proved very rich in cultivable bacteria on LB and, particularly, R2A media (Figure 1). A large diversity of colony phenotypes was observed at temperatures from 4°C to 27°C, with very few cultivable isolates growing at higher temperatures (50°C). Many of the isolates displayed yellow, orange or pink colors, particularly on R2A. In fact, R2A plates incubated at temperatures from 4°C to 27°C displayed numerous pink-pigmented colonies.

Due to the diverse microbial growth observed on the R2A plates, this media was selected for all the further studies and isolates were re-streaked exclusively from R2A plates. Specifically, seven isolates from the R2A plates grown at RT (SPB1-SPB7) were randomly selected along with three pink-pigmented isolates from the R2A plates grown at 4°C (SPB8-SPB10). Additionally, four isolates previously selected from the solar panels samples by UV irradiating for 5 min (as described in Materials and Methods) were selected as well (data not shown) (SPB11-SPB14). In total, 14 isolates were identified by 16S rRNA gene sequencing as follows: *Arthrobacter* (SPB1), *Hymenobacter* (SPB2), *Hymenobacter* (SPB3), *Rhodococcus* (uranium-contaminated site) (SPB4), *Methylobacterium* (SPB5), *Deinococcus* (SPB6), *Arthrobacter agilis* (SPB7), *Hymenobacter* (SPB8), *Hymenobacter* (SPB9), *Hymenobacter perfusus*-uranium (SPB10), *Hymenobacter perfusus*-uranium (SPB11), *Curtobacterium* (SPB12), *Curtobacterium* (SPB13), and *Arthrobacter agilis* (SPB14).

**FIGURE 2:**
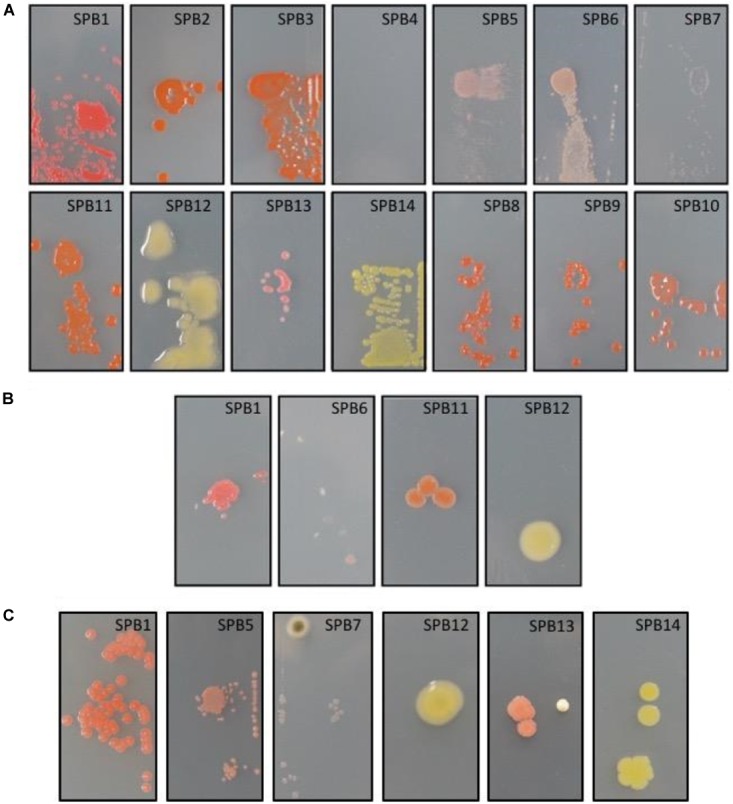
**(A)** Glass-adhesion test performed as described in M&M. From left to right, top: isolates SPB1, SPB2, SPB3, SPB4, SPB5, SPB6, SPB7; bottom: SPB11, SPB12, SPB13, SPB14, SPB8, SPB9, and SPB10. **(B)** UV-resistance test performed on glass-adhering cells as described in M&M. From left to right isolates SPB1, SPB6, SPB11, and SPB12. The three later correspond to the growth of only 1-3 UV-resistant colonies each. **(C)** Desiccation-resistance test performed on glass-adhering cells as described in M&M. From left to right isolates SPB1, SPB5, SPB7, SPB12, SPB13, and SPB14. Isolates correspond to: SPB1, *Arthrobacter*; SPB2, *Hymenobacter*; SPB3, *Hymenobacter*; SPB4, *Rhodococcus* (uranium-contaminated site); SPB5, *Methylobacterium*; SPB6, *Deinococcus*; SPB7, *Arthrobacter agilis*; SPB8, *Hymenobacter*; SPB9, *Hymenobacter*; SPB10, *Hymenobacter perfusus-uranium*; SPB11, *Hymenobacter perfusus-uranium*; SPB12, *Curtobacterium*; SPB13, *Curtobacterium*; and SPB14, *Arthrobacter agilis*. The images are representative of the microscope slides (size 22X40 mm).

The 14 isolates were then screened for their glass-colonization abilities. After 2 days of incubation, strains SPB1, SPB5, and SPB6 exhibited very faint but visible colonies. After 4 days, all but one strain were able to grow, indicating some adhesion ability to the glass surfaces (Figure 2A). The strains with the highest glass colonization ability, as deduced by a fully compact growth on the slide were SPB1, SPB5, and SPB6, and to a lesser extent, SPB11 and SPB3 (Figure 2A). When subjected to 2 min of UV irradiation, only one strain (SPB1), exhibited high resistance as deduced by numerous colonies (>10) growing after transfer to R2A solid medium (Figure 2, left). Three other isolates resulted ≤ three colonies each (SPB6, SPB11 and SPB12) and the rest of isolates did not yield viable cells after irradiation (Figure 2B).

As it was the case with UV radiation, 72 h desiccation tests yielded a decrease in viability of most of the strains. Only strain SPB5 exhibited vigorous growth, concentered around the spot on which the suspension was placed; followed by SPB1, with hundreds of surviving colonies. The remaining isolates exhibited very low (<20 colonies for SPB7, 12, 13, 14) to no survival to desiccation (Figure 2C).

### Metagenomic Analysis

**FIGURE 3:**
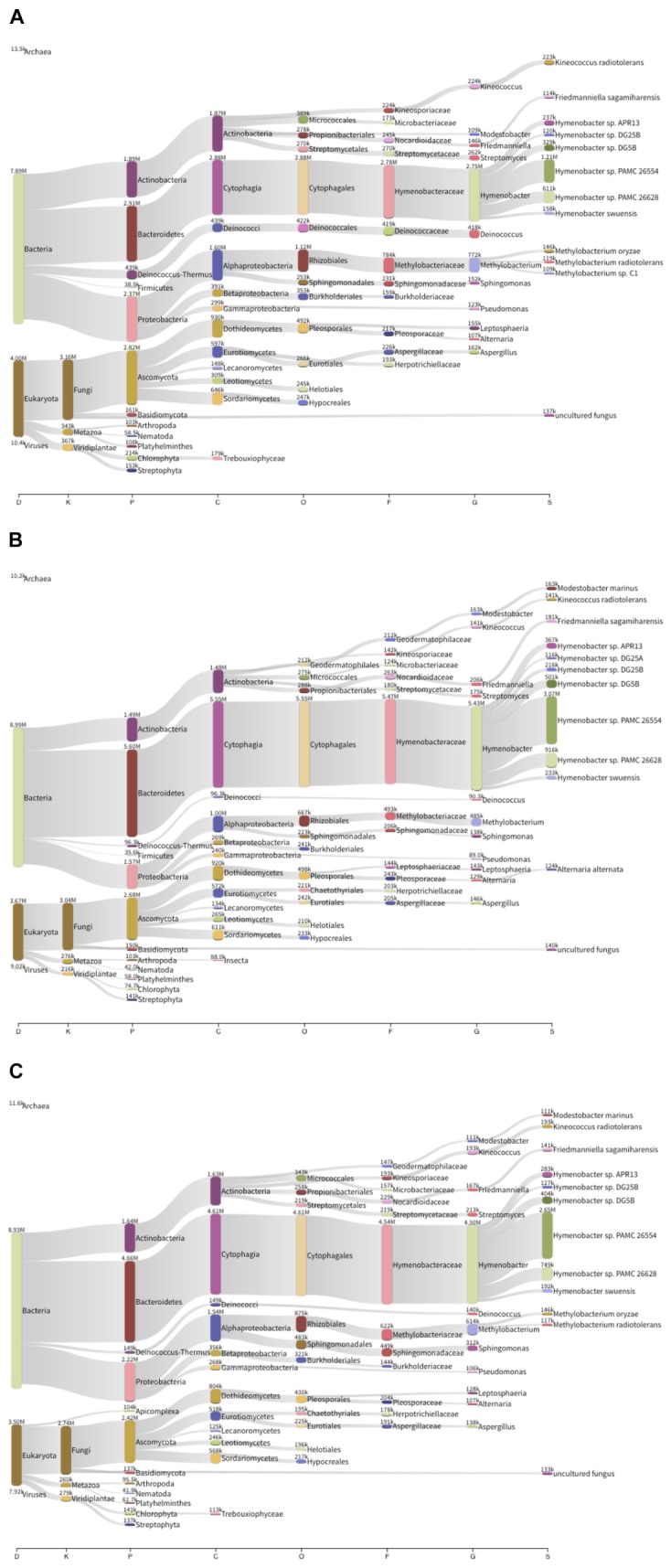
Taxonomic composition of three solar panel microbial communities from Berkeley, CA, United States. The thickness of the lines is representative of the relative abundance of the taxa. **(A)** Left solar panel. **(B)** Center solar panel. **(C)** Right solar panel.

Between 590 and 775 Mb were sequenced for each sample and assembled into around 710.000 and 1 million scaffolds. Approximately one million ORFs were predicted for each metagenome: 99.11% of the ORFs corresponded to protein-coding genes, and the remaining 0.89% to RNA genes. Taxonomic analysis (Figure 3) revealed that the sequences corresponded mainly to bacteria, although there was also a substantial proportion of eukaryota, in which predominant sequences corresponded to fungi and, more specifically, to Ascomycota (∼31.9% of annotated contigs across the three metagenomes). In the case of bacteria, the predominant phyla were Actinobacteria (15.6%), Bacteroidetes (22.6%), and Proteobacteria (14.8%), and to a lesser extent, Deinococcus (6.3%) Cyanobacteria and Firmicutes. Furthermore, there was a clear predominance of *Hymenobacter* spp. amongst the microbial community of the Berkeley solar panels (19.7%), with other constituents including *Deinococcus* spp. (6.3%), *Modestobacter marinus* (1.25%), *Kineococcus radiotolerans* (3.13%), *Friedmanniella sagamiharensis* (4.98%) and *Alternaria alternata* (2.19%), among others. The results of our metagenomic sequencing clearly support our culture-based approach, as all our cultured isolates are represented in our assembled metagenomes.

**FIGURE 4:**
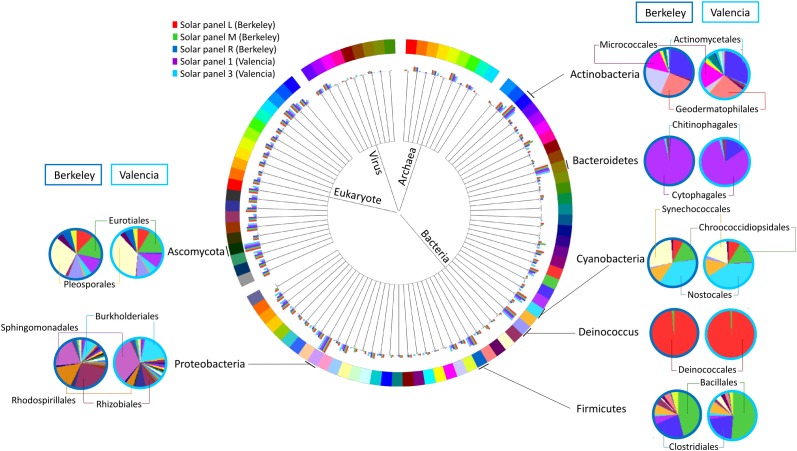
Comparison of the taxonomic profiles of solar panels from Berkeley, CA, United States (red, green, and dark blue bars – three replicates) and Valencia, Spain (purple and light blue bars – two replicates). Most abundant taxa are indicated, and subdivisions of those taxa in one replicate from each location are represented (Berkeley and Valencia replicates in the dark and light blue circles, respectively).

**FIGURE 5:**
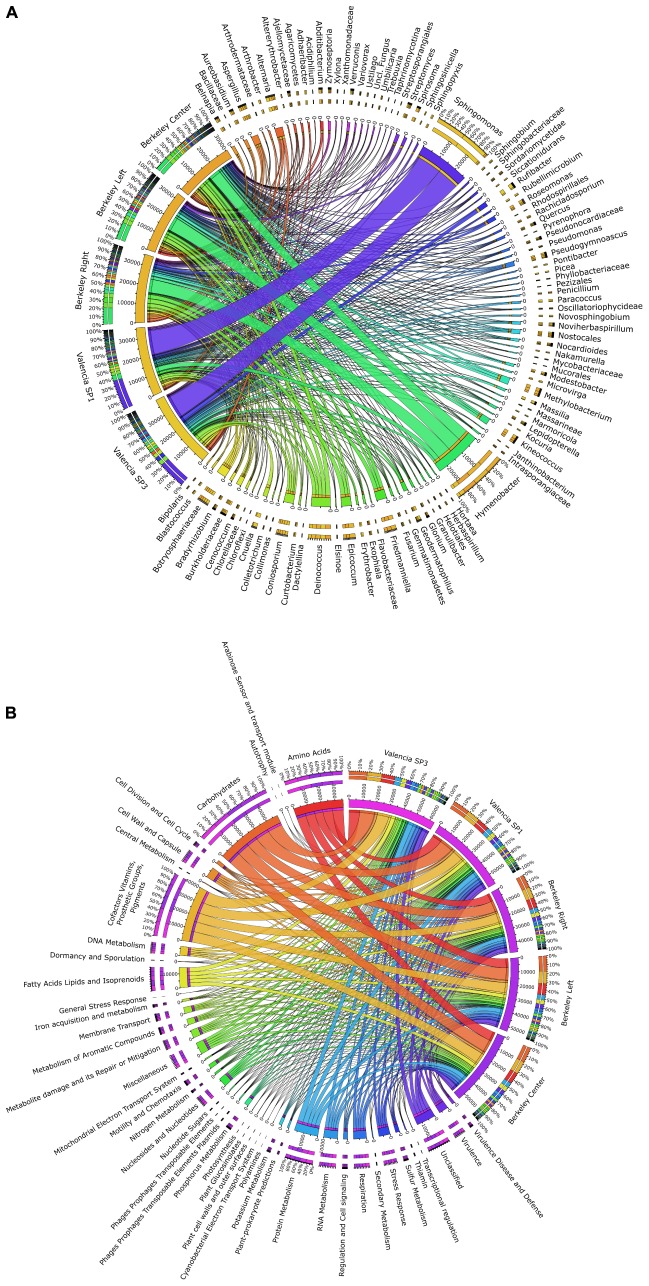
Circos graph connecting **(A)** microbial taxa at the genus level and **(B)** SEED functional subsystems to the different metagenomes analyzed in this work (three solar panels from Berkeley, CA, United States, and two solar panels from Valencia, Spain).

When comparing the taxonomic information of the solar panels from Berkeley with the data obtained from solar panels in Valencia (Dorado-Morales et al., 2016), the taxonomic profiles proved very similar both in community composition and taxon abundance (Figure 4). Specifically the most abundant taxa in all five samples were Actinobacteria, Bacteroidetes (mainly Cytophagales), Cyanobacteria, Deinococcus (mainly Deinococcales), Firmicutes, Proteobacteria and Ascomycota; and the subdivisions of these taxa were very similar in the solar panels from both locations (Figure 4). Despite these general similarities, we found a number of significant differences between localities at various taxonomic levels. Specifically, members of the Ascomycota and Bacteroidetes were significantly enriched in the Berkeley samples compared to the Valencia communities (Welch’s two-sided *t*-test, *P* < 0.05). By contrast, Alphaproteobacteria were significantly more common in the Valencia metagenomes than the Berkeley counterparts (*P* < 0.05), as were *Sphingomonas* spp. (*P* < 0.05) (Figure 5A). Statistical analyses indicate that these communities differ significantly in their composition according to sampling location (Valencia vs. California; PERMANOVA, *P* < 0.001).

Consistent with the observed taxonomic variations between the solar panel communities, we found marked differences in the functional attributes of the solar panel communities. Firstly, comparisons of our genes against the SEED subsystems database (Figure 5B) showed that pathways involved in the persistence of microbes on solar panels, such as stress response (3.1% of annotated open reading frames), capsule development (2.8%) and metabolite repair (2.1%), were common to all metagenomes. We also found evidence of genes for carotenoid biosynthesis and, by contrast, genes assigned to photosynthetic pathways were rare (0.07%) as were those assigned to general autotrophic subsystems (0.02%) suggesting that these pathways are not critical for persistence on solar panels.

Notwithstanding these dominant processes, we found significant over-representation of catalases, cAMP-binding proteins and 3-oxoacyl-[acyl-carrier protein] reductases in the Berkeley metagenomes compared to the Valencia samples (Welch’s two-sided *t*-test, *P* < 0.05). The opposite trend was observed for DNA-dependent RNA polymerases and TonB-dependent receptors (*P* < 0.05), which were more abundant in the metagenomes from Valencia. Cumulatively, the differences in gene content between the communities were sufficient to explain >70% of the variation between the metagenomes collected from Valencia and Berkeley (PCA, First principal component = 70.7%; PERMANOVA, *P* < 0.05).

A more targeted analysis of the functional components of these metagenomes revealed diverse mechanisms for dealing with the extreme climatic conditions imposed by living on solar panels. We found numerous genes encoding heat shock chaperone proteins (e.g., *dnaK, dnaJ, grpE*; combined genes across Berkeley metagenomes, *n* = 187) which belonged to a range of taxa, but were primarily affiliated with *Deinococcus* spp. and *Sphingomonas* spp. Mechanisms of combatting oxidative stress were equally abundant in both locations and included a variety of superoxide dismutases (*n* = 50), most of which belonged to *Kineococcus radiotolerans* and *Deinococcus* spp., as well as a group of peroxidases and peroxide stress regulators, which were assigned exclusively to members of the *Methylobacteria*. Perhaps the most ubiquitous stress responses were those involved in DNA damage repair which provided between 459 and 519 genes per metagenome. DNA mismatch repair genes *mutL* and *mutS* were very common features within the metagenomes and could be assigned to a diverse set of dominant bacterial groups including *Hymenobacter* spp. and *Sphingomonas* spp., among others.

Finally, our functional data strongly corroborate our metabolomics results (described in the section below). Pathways for allantoin utilization were common to all metagenomes and include allantoinase and allantoicase, two hydrolase families involved in the biogenesis and degradation of ureides. As observed in the metabolomics data, we found more genes involved in allantoin metabolism in the Berkeley samples than in the Valencian samples. For example, allantoate amidohydrolase and allantoin racemase were present exclusively in the Berkeley metagenomes. These processes appear to be carried out by both dominant (i.e., *Deinococcus* spp.) and rare (i.e., *Thermobispora bispora*) community members, indicating a widespread gene catalog for key processes that permit colonization in an extreme environment.

### Metabolomics Results

**FIGURE 6:**
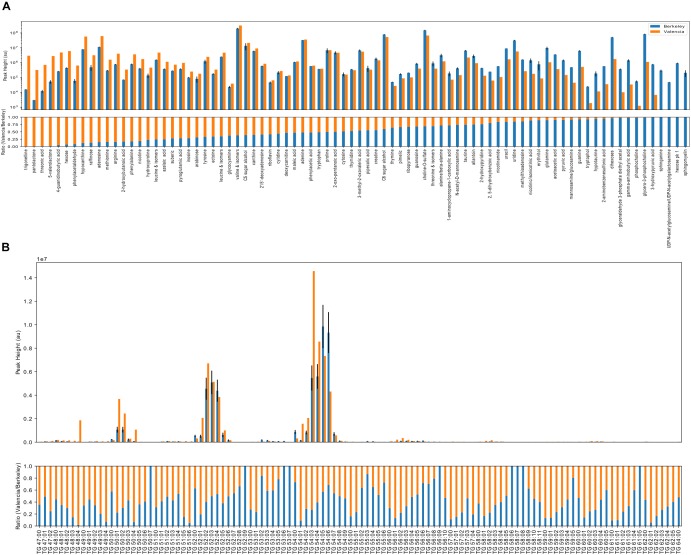
Metabolomics analyses of the Californian and Valencian solar panel samples. Ion abundance results for polar metabolites **(A)** and triacylglycerol lipids **(B)**. Absolute ion abundances (upper panels, log scale) and relative ion abundances (lower panels, scaled to 1) corresponding to the identified metabolites are indicated in orange (Valencia, Spain) and blue (Berkeley, CA, United States). Without quantification, ion abundances cannot be used to compare between metabolites due to differences in ionization efficiencies. Here, ion abundances of identified metabolites may be used to compare relative abundances between Berkeley and Valencia. Polar metabolites **(A)** are ranked by relative abundance and triacylglycerol lipids are sorted by chain length followed by degree of unsaturation.

Most of the detected polar metabolites were present in both locations, although a few were detected primarily in a single location (Figure 6A). In both locations, common primary metabolites such as amino acids, nucleobases and sugars were detected. Interestingly, both locations contained nicotine, which may be linked to outdoor smoking. A number of aliphatic dicarboxylic acids of variable chain lengths (maleic acid, azelaic acid, suberic acid, pimelic acid) were present in both. Compatible solutes, such as ectoine, sugar alcohols, di- and tri-saccharides, were detected in both, which may play a role in protection against desiccation, heat and/or UV stress. A few compounds, sphinganine, sphingomyelin, an unidentified hexose and UDP-acetylhexosamine were detected only in the Berkeley samples while trigonelline, pantolactone, 5-valerolactone, and threonic acid and 4-guanidinobutyric acid had higher relative abundance in the Valencian sample. Triglyceride (TG) metabolites were highly abundant in both locations, and the most abundant triglycerides were similar between both locations (Figure 6B). The metagenomic and metabolomic data are publicly available in the JGI database under accession number ID: 503162, and can be accessed with the following URL: https://genome.jgi.doe.gov/portal/solcelcoanalysis/solcelcoanalysis.info.html.

## Discussion

Samples isolated from solar panels in Berkeley, CA, United States proved very rich in culturable bacteria despite the harsh environmental conditions they are subjected to, a result that is consistent with the previous work done on solar panels from Valencia, Spain and polar regions (Dorado-Morales et al., 2016; Tanner et al., 2018). Interestingly, the vast majority of the culturable microorganisms were not thermotolerant, but mesophilic or even psychrotolerant. This has important implications for the ecology of an environment that is prone to have thermal stress and daily peaks of extreme heat, particularly in summer (Dorado-Morales et al., 2016), when sampling was performed. Taking into account that peaks of heat on the panels tend to correlate with drought, our results suggest that microbial growth may be concentrated during the night, when water availability is higher and temperatures much cooler, even in Mediterranean climates. The average low temperature in Berkeley in August is just 12.4°C (Western Regional Climate Center, accessed May 5th 2017). This preference for mild growth temperatures was also observed in the isolates from Spain (Dorado-Morales et al., 2016). Taken together, both reports strongly suggest a thermoresistant -but not thermophilic- solar panel-adapted community.

Growth on both LB and R2A media yielded a large proportion of pigmented colonies. Interestingly, the highest number of pigmented colonies was observed when the samples were grown on R2A medium at 4°C, which could be explained by the increased accumulation of carotenoids at low temperatures as a cryoprotection strategy through the modulation of membrane fluidity (Jagannadham et al., 2000; Dieser et al., 2010). Furthermore, growth of *Hymenobacter* on R2A medium and at temperatures between 4 and 25°C is consistent with previous reports (Srinivasan et al., 2015; Lee et al., 2016) and suggests the preference of this bacterium for low nutrient culture media. The well-known role of carotenoids as UV sunscreens and the abundance of pigmented strains in panels strongly suggests their involvement in radiation protection during the day.

Besides heat-, radiation- and drought-resistance, microorganisms living on a smooth, flat surface fully exposed to the harsh climate must firmly attach to the substrate. Such attachment can involve binding to dust and other inorganic particles, but at least in the first stages of colonization, strong adhesion to the glass surface is likely to be a major selective force. In order to characterize the glass-adhesion abilities as well as the resistance to UV light and desiccation of glass-bound cells, we developed an *ad hoc* test for some of the culturable strains from the solar panels. As expected, almost all of the isolated strains tested positive for adhesion to glass, with the exception of *Rhodococcus*, which is surprising taking into account that this genus typically produces extracellular polysaccharides that have a role in adhesion to surfaces (Urai et al., 2007). On the other hand, UV-radiation experiments resulted in the selection of only four UV-resistance isolates under our conditions (*Arthrobacter* spp., *Deinococcus* spp., *Hymenobacter* spp., and *Curtobacterium* spp.), whose extreme radiation-resistance properties have previously been reported (Jacobs and Sundin, 2001; Mongodin et al., 2006; Chung et al., 2010; Gerber et al., 2015). The lack of a higher number of UV-resistant isolates from a highly irradiated source environment is intriguing, and it could be explained by the effect of dust or sub-aerial biofilms shadowing on bacteria, thus mediating survival of low-resistant organisms (Osman et al., 2008). Desiccation experiments on glass revealed *Arthrobacter* and *Methylobacterium* as the most resistant isolates, consistent with previous reports concerning the desiccation-resistance properties of these two genera (Makhalanyane et al., 2013; SantaCruz-Calvo et al., 2013).

These results suggest that sun-exposed surfaces such as solar panels can be rich reservoirs of biotechnologically interesting bacteria thanks to their adhesion, radiation-resistance and desiccation-resistant properties, as well as to the production of sunscreens and/or antioxidant compounds such as carotenoids. This potential could of course increase when considering the non-culturable fraction of the sampled microbiomes. In order to further characterize the solar panels from California, a culture-independent approach combining metagenomic sequencing and metabolomics was set in place.

High-throughput sequencing of the solar panel samples revealed that these structures are composed of a rather diverse microbial population. In concordance with the culture-based characterization described above, the microbiome was dominated by *Hymenobacter* spp. and, to a lesser extent, by well-known radiation-resistant organisms, such as: *Modestobacter marinus*, an *Actinobacterium* that grows on calcareous stone surfaces (Normand et al., 2012); *Kineococcus radiotolerans*, previously isolated from radioactive areas (Phillips et al., 2002); or *Alternaria alternata*, a plant pathogenic fungus also found to grow inside the Chernobyl reactor (Mironenko et al., 2000).

Regarding the metabolomics analysis, although most of the detected polar compounds are common intracellular metabolites, a few were differentially expressed between the two locations. For example, trigonelline, a thermally labile secondary metabolite that is present in leguminous and, to a lesser extent, non-leguminous plants (Ashihara and Watanabe, 2014), as well as mammal urine, have been shown to inhibit attachment of bacteria to surfaces (Daglia et al., 2002). There have been previous reports on the ability of rhizosphere microorganisms to perform trigonelline catabolism (Boivin et al., 1991; Goldmann et al., 1991), but there are no reports (to the best of our knowledge) of microorganisms able to produce trigonelline. Pantolactone, 5-valerolactone, threonic acid and 4-guanidinobutyric acid were >10-fold more abundant in the Valencia sample. Threonic acid is a product of ascorbic acid metabolism (vitamin C), a well-known antioxidant compound; the degradation of ascorbic acid has been described in a variety of bacteria, including *Lactobacillus* spp., a genus detected in the Valencian sample (Englard and Seifter, 1986; Montaño et al., 2013). On the other hand, 5-valerolactone in an intermediate in the metabolism of cyclopentanone, a pathway that has been previously described in *Pseudomonas* spp. (Griffin and Trudgill, 1972) and *Comamonas* spp. (Iwaki et al., 2002). Interestingly, dye-sensitized solar cells have been previously fabricated with 4-guanidinobutyric acid as co-adsorbent, leading to an approximately 50 mV increase in open-circuit voltage in comparison to cells without GBA cografting (Zhang et al., 2005). This molecule could also be present due to conversion from L-arginine by means of the L-arginine oxidase, an enzyme that has been previously described in *Pseudomonas* spp. (Matsui et al., 2016) and cyanobacteria (Schriek et al., 2007). Compounds including sphingomyelin, sphinganine, N-acetylhexosamine and were only detected in the Berkeley, CA, United States samples. Sphingomyelin is the most frequently occurring mammalian sphingolipid, although it has previously been described in *B. thetaiotaomicron* (Olsen and Jantzen, 2001). Interestingly, sphinganine has proven to inhibit bacterial adherence and to negatively affect biofilm formation in *Staphylococcus aureus, Streptococcus mitis* and *Streptococcus mutans* (Bibel et al., 1992; Cukkemane et al., 2015). N-acetylglucosamine, is an important component of the bacterial and fungal cell walls, and along with insect chitin may play a signaling role across multiple kingdoms (Konopka, 2012).

There were also a few metabolites detected from both Valencia and Berkeley that were of special interest given the environmental conditions on the solar panels. Interestingly, a number of medium chain length dicarboxylic acids were detected in samples from both Berkeley and Valencia. Only a single transporter was found in the metagenome for a short chain dicarboxylic acid. Azelaic acid, a bactericidal agent produced in fungi, plants and animals, can also be utilized as a sole carbon source by Burkholderia spp. (Estrada-de los Santos et al., 2001), a genus identified in both Berkeley and Valencia samples. The presence of compatible solutes in both locations is not surprising given the exposure to high heat and UV irradiation. Ectoine and 5-hydroxyectoine are produced by bacteria for protection against osmotic stress and more recently have been demonstrated to protect mammalian DNA from UV damage (Czech et al., 2018); however, only a single gene, assigned to Bradyrhizobium, was found in the Valencia, Spain metagenome for production of 5-hydroxyectoine from ectoine. Polyols, many of which were present in both Berkeley and Valencia samples, accumulate in yeasts in response to osmotic stress (Tekolo et al., 2010). Thus it was not surprising that numerous genes involved in polyol and trehalose biosynthesis, utilization and degradation were detected across a diverse set of bacteria. Pipecolic acid, a precursor to secondary metabolites, is produced in both bacteria and fungi (He, 2006). Allantoin is utilized by some bacteria as a secondary source of nitrogen under nutrient-limiting conditions (Ma et al., 2016). Tryptophol may act as a signaling molecule and precursor to secondary metabolites in fungi and yeasts (Palmieri and Petrini, 2018).

Triglyceride metabolites were detected in both locations, and this is not surprising, given that cells enduring an environmental stress such as desiccation (as found on a solar panel) often shift metabolic energy to a more quiescent state and toward carbon storage, e.g., TG accumulation and fatty acid storage in TGs (Rittershaus et al., 2013). The most abundant triglycerides were similar among both locations, and this may be attributed to the strikingly similar taxonomic profiles of the solar panels between Spain and California (Figure 4), since lipid composition is characteristic of species and often similar between species from the same taxa (Sohlenkamp and Geiger, 2016).

As recently described for solar panels in the North and South Poles (Tanner et al., 2018), there is also a striking similarity between the taxonomic and functional profiles from solar panels from two same-latitude locations: Berkeley, CA, United States and in the distant Mediterranean city of Valencia, Spain (Figure 4). This is certainly related to the common environmental conditions, including the climate and the selective pressures associated to a fully sun-exposed habitat on a glass surface: thermal fluctuations and heat peaks, high irradiation and circadian cycles of wetting and desiccation. These common stressors, which also include limited C and N availability, have created communities that are strikingly similar in terms of their functional capacity (Figure 5B), even though we observed nuanced differences for some essential processes. This indicates a high degree of functional redundancy, whereby the variety of stress response adaptations occur in multiple individual microbial groups within each community. Although common selective pressures are expected to yield adaptive convergence, as observed in our results, rather than a taxonomic similarity, the comparison between the Valencian and Berkeley solar panels strongly suggests that, besides the climate, there must be similar inocula involved in the colonization process (Figure 5A). As previously reported elsewhere, the wind is a major source of air-borne bacteria (Hervàs et al., 2009; Barberán et al., 2015; Meola et al., 2015), which, along with birds, insects and other animals, might be the main source of inocula for the solar panel microbiome to develop. Our results are in concordance with a world-wide distribution of bacterial diversity, which is shaped *in situ*, by the specific pressure of living on a solar panel.

## Author Contributions

MP and TN designed the project. MP, KL, and SK performed the experiments. MP, BB, KL, SK, MG, and KT analyzed the data. MP, KL, SK, BB, KT, MG, and TN wrote and revised the manuscript. TN and MP provided the funding.

## Conflict of Interest Statement

The authors declare that the research was conducted in the absence of any commercial or financial relationships that could be construed as a potential conflict of interest.
